# The Microbiome of Prostate Fluid Is Associated With Prostate Cancer

**DOI:** 10.3389/fmicb.2019.01664

**Published:** 2019-07-19

**Authors:** Xiaowei Ma, Chenfei Chi, Liancheng Fan, Baijun Dong, Xiaoguang Shao, Shaowei Xie, Min Li, Wei Xue

**Affiliations:** ^1^Department of Urology, Renji Hospital, School of Medicine, Shanghai Jiao Tong University, Shanghai, China; ^2^Department of Clinical Laboratory, Renji Hospital, School of Medicine, Shanghai Jiao Tong University, Shanghai, China; ^3^Department of Ultrasound in Medicine, Renji Hospital, School of Medicine, Shanghai Jiao Tong University, Shanghai, China

**Keywords:** prostate cancer, prostatic fluid, microbiome, cancer, prostate

## Abstract

**Objectives:**

To explore the microbiome of the prostatic fluid in high prostate-specific antigen (PSA) patients.

**Patients and Methods:**

The microbiome profiles of prostatic fluid samples from 32 prostate cancer (PCa) patients and 27 non-PCa people were assessed. Microbiome analysis was assessed by massive 16S ribosomal RNA gene sequencing.

**Results:**

Compared with the NCA group, the microbial diversity was lower in the CA group. There were no specific microbial species in the CA group or NCA group. However, many species, such as those in the genera *Alkaliphilus*, *Enterobacter*, *Lactococcus*, *Cronobacter*, *Carnobacterium*, and *Streptococcus*, showed a significant difference between the CA group and NCA group.

**Conclusion:**

The prostate contains reduced bacteria, suggesting a possible pathophysiological correlation between the composition of the microbiome and PCa. Meanwhile, this study uncovered that the microbiome may be beneficial in maintaining the stability of the microenvironment of the prostate and provides interesting perspectives for the identification of novel biomarkers in high-PSA patients.

## Introduction

Prostate cancer (PCa) is one of the commonest male malignant tumors, and it is the second most diagnosed malignant tumor and the fifth leading cause of tumor-associated death in the world (Wong et al., 2016). At the moment, PCa is the third highest cause of tumor-associated mortality in the United States (Siegel et al., 2017). In addition, the incidence of PCa shows, recently, a trend of fast increase in Asian countries, including China (Bray et al., 2010). Moreover, PCa seriously influences sexual functions, male urinary and decreases the quality of life (Chipman et al., 2014). Despite great efforts made worldwide to explore the novel treatment strategies of PCa, the patients generally relapse and develop resistance in advanced stages. Therefore, we should draw great attention to and search for better approaches for the diagnosis and treatment of PCa.

Prostate-specific antigen (PSA), an enzyme secreted by the prostate gland, increases in the blood of patients with PCa; therefore, it is widely used as an established laboratory test for PCa. However, as it is commonly used to diagnose PCa, misdiagnosis events frequently occur due to its high sensitivity and low specificity, and its value in screening, particularly in asymptomatic males, is controversial when considering the risks and benefits of early detection. Tumors and inflammation can both lead to damage to the prostate gland, causing increased levels of PSA.

Chronic prostatitis (CPS), a common chronic inflammation disease in adult males, accounts for more than 90%. In addition, studies have shown that chronic inflammation in the prostate has been especially relevant for the progression, pathogeny, and prognosis of PCa (Taverna et al., 2015; He et al., 2017), and CPS (Giunchi et al., 2017) is one of the causes of elevated PSA. According to different inflammatory and tumor conditions in previous researches, the modifications of bacterial populations were also founded in PCa samples and partly promote the development of cancer by enhancing the pro-inflammatory responses or changing the extracellular environment of the prostate (Cohen et al., 2005; Shinohara et al., 2013; Cavarretta et al., 2017). It has been reported that many pathogenic microorganisms could induce symptomatic asymptomatic and symptomatic inflammatory reactions in the prostate, including *Escherichia coli* (Buerfent et al., 2015), *Pseudomonas* spp. (Souto et al., 2014), *Neisseria gonorrhoeae* (Churchward et al., 2017), *Chlamydia trachomatis* (Koroleva et al., 2015), and *Trichomonas vaginalis* (Lee et al., 2012). In addition, *Propionibacterium acnes*, detected in PCa patients’ samples through direct genomic amplification and culture, has been associated with enhanced inflammatory response in patients with PCa. Now that CPS and PCa have been closely associated with the microbiome, revealing the connection in the microorganisms associated with PCa and CPS might be important.

Until now, a comprehensive and detailed comparison of the microbial ecosystems of the prostatic fluid of PCa patients and non-PCa people has not been conducted. Therefore, the objectives of the current study are to characterize the microbiomes associated with the non-tumor and tumor prostatic fluid microenvironment in high-PSA people using 16S ribosomal RNA (rRNA) gene sequencing and assess their relevance in terms of the pathogenesis of PCa as well as to find a connection in the microorganisms associated with PCa and CPS with the hope to seek out a new means for the diagnosis of PCa.

## Patients and Methods

### Patient Selection and Specimen Processing

From May 2015 to October 2016, prostatic fluid specimens from 59 patients who had a high level of PSA (>4 ng/ml) before prostate biopsy were chosen for our study. None of the patients had recent urinary tract infections or sexually transmitted infections. Patients with a pathologic International Prostate Symptom Score (IPSS) or lower urinary tract symptoms (LUTS) were excluded. Nobody have any antimicrobial exposure within the preceding 4 week. The diagnosis of 32 PCa patients and 27 non-PCa people was determined by pathology after prostate biopsy. Data collection was conducted according to the principles outlined in the Declaration of Helsinki. All participants signed an informed consent form agreeing to provide their own anonymous information for future research. The study was approved by the ethical committee of the Renji Hospital (Renji/2013126). Table 1 details the clinical characteristics and pathological parameters of the participators.

**TABLE 1:** The clinical characteristics and pathological parameters of the two groups.

	**CA (*n* = 32)**	**NCA (*n* = 27)**	***P***
Age (years)	68.21 ± 1.04	65.00 ± 1.67	0.0516
WBC (10^9^/L)	5.91 ± 0.43	6.87 ± 0.27	0.0594
PSA (ng/μl)	17.48 ± 2.60	31.72 ± 15.67	0.5866
fPSA/PSA	9.12 ± 1.87	13.57 ± 1.36	0.0679
Gleason 6	43.75% (14/32)		
Gleason 7	46.8% (15/32)		
Gleason 8	9.4% (3/32)		

*CA, prostate cancer; NCA, non-prostate cancer; PSA, prostate specific antigen; fPSA, free prostate specific antigen; Gleason, Prostate Gleason Grading system; WBC, white blood count.*

### Sample Collection and DNA Extraction

Prostatic fluid samples were collected at the hospital through massaging the prostate before prostate biopsy under sterile acquisition and stored at −80°C within 1 h. DNA extraction was performed using a QIAamp DNA Mini Kit (Qiagen, Valencia, CA, United States). The concentration of bacterial DNA was measured using a Nanodrop 2000 (Thermo Fisher Scientific, United States). Data on demographics and clinical variables were collected during the clinic visits.

### 16S Ribosomal RNA Gene Sequencing

The V3–V4 region of the bacterial 16S rRNA gene was amplified using PCR with the barcode-indexed primers 338F and 806R using a PCR thermocycler system (GeneAmp 9700, ABI, United States). A negative control was simultaneously amplified. If the negative control was negative, the resulting PCR products were extracted from a 2% agarose gel and further purified using the AxyPrep DNA Gel Extraction Kit (Axygen Biosciences, Union City, CA, United States) and quantified using QuantiFluor^TM^-ST (Promega, United States) according to the manufacturer’s protocol. The purified amplicons were pooled in equimolar concentrations, and paired-end sequencing (2 × 300) was performed using an Illumina MiSeq instrument (Illumina, San Diego, CA, United States).

### Processing of the Sequencing Data

Raw FASTQ files were demultiplexed, quality-filtered with Trimmomatic and merged using FLASH with the following criteria: (i) the reads were truncated at any site receiving an average quality score <20 over a 50 bp sliding window. (ii) Primers were exactly matched, allowing two nucleotide mismatching, and reads containing ambiguous bases were removed. (iii) Sequences whose overlap was longer than 10 bp were merged according to their overlap sequence.

Operational taxonomic units (OTUs) were clustered with a 97% similarity cutoff using UPARSE (version 7.1^1^), and chimeric sequences were identified and removed using UCHIME. Then, the singletons were also removed. The taxonomy of each 16S rRNA gene sequence was analyzed using the RDP Classifier algorithm^2^ against the Silva (SSU123) 16S rRNA database using a confidence threshold of 70%. OTUs with a number of sequences <0.005% of the total number of sequences were removed from the OTU table. In addition, rarefaction was performed on the OTU table to prevent methodological artifacts arising from varying sequencing depths. α-Diversity was measured by species richness from the rarefied OTU table. β-Diversity was estimated by computing the unweighted UniFrac distance and was visualized using principal coordinate analysis. In an effort to identify the possible species represented by the OTUs, we performed a MegaBLAST search to align the reads of the OTUs against reference sequences in the National Center for Biotechnology Information (NCBI) 16S rRNA database. The data has been uploaded to SRA database (SRP197683).

### Statistical Analysis

All statistical analyses were performed using R packages (V.2.15.3) and SPSS 16.0 (SPSS Inc., Chicago, IL, United States). For the comparison of continuous variables, data are presented as the median (first quartile to the third quartile). The statistical significance of the differences among different groups was tested using Friedman’s test, with the Wilcoxon rank-sum test for CA (cancer group) versus NCA (non-PCa) (according to the data distribution). For the correlation analysis, Spearman’s rank test was performed. Multiple hypothesis tests were adjusted using the Benjamini and Hochberg false discovery rate (FDR), and a significant association was considered to occur when the FDR was below the threshold of 0.05. A random forest model (randomForest 4.6-7 package) using the 12-genera signature was applied for the data from the PBC and control samples. To evaluate the discriminatory ability of the random forest model, operating characteristic curves (receiving operational curve, ROC) were constructed, and the area under the curve (AUC) was calculated.

## Results

### Sequencing Data Quality Analysis

**FIGURE 1:**
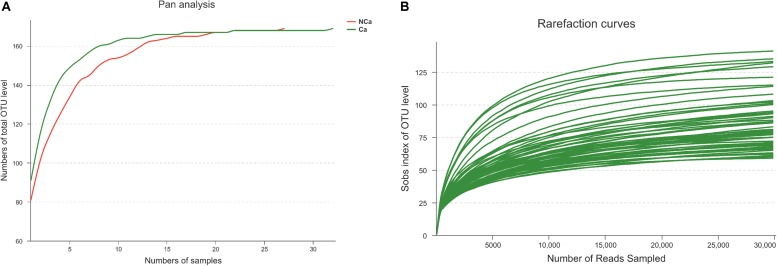
Prostate 16S ribosomal RNA gene sequencing data analysis. **(A)** Number of total OTUs in all samples. **(B)** The Sobs index of the OTU levels in all samples.

Prostatic fluid from 32 PCa patients and 27 non-PCa (NCA) people was submitted to 16S rRNA gene sequencing. The pan analysis was smooth and steady after 20 samples, indicating that the number of samples was sufficient for the analysis (Figure 1A). The rarefaction curves of all samples were smooth and steady (Figure 1B), indicating that the depth of sequencing was sufficient for the diversity analysis.

### PCa Samples Showed Reduced Microbial Diversity

**FIGURE 2:**
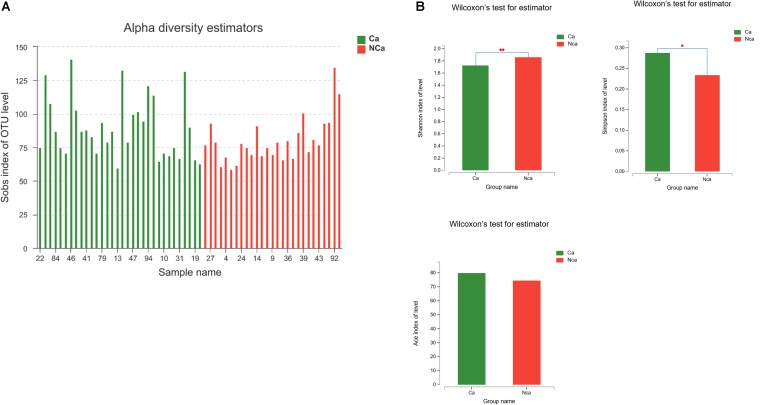
PCa samples showed reduced microbial diversity. **(A)** The alpha microbial diversity presented in all samples. **(B)** The Shannon–Wiener index, Simpson index, and Ace index in the CA group and NCA group.

**FIGURE 3:**
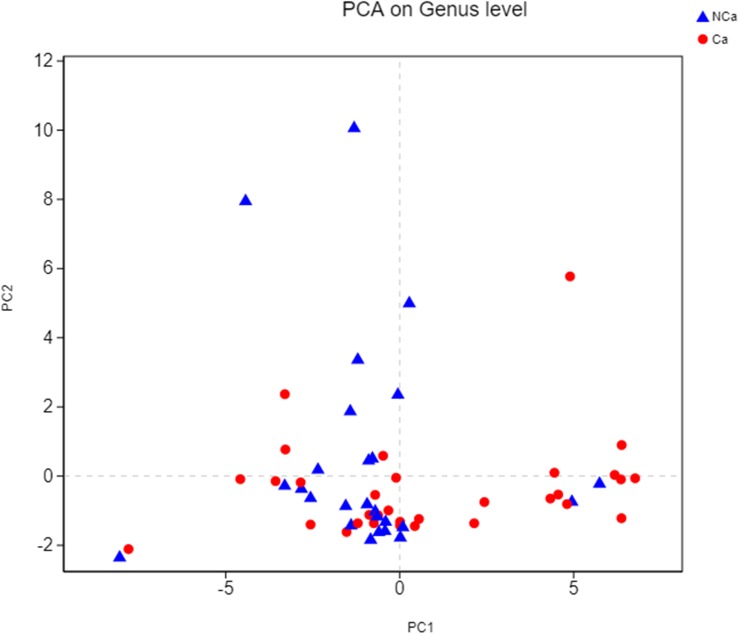
PCA on genus level.

Then, we compared the microbial alpha diversity of the two groups (the CA group represented samples from the PCa patients, and the NCA group represented the samples from non-PCA people) by calculating the Shannon–Wiener, Simpson and Ace diversity indexes. The alpha diversity was determined based on community richness and community evenness. There did not appear to be a difference in the Sobs index for the OTU level between the two groups in Figure 2A. Therefore, the Ace index showed no difference in community richness between the two groups. Meanwhile, the difference in the Shannon–Wiener and Simpson indexes in Figure 2B indicated that there was a significant difference in community evenness between the two groups. The data demonstrated reduced microbial diversity in the prostatic fluid from people with PCa. The PCA analysis at the genus level showed that the NCA and CA groups had some clustering with members of their own group (Figure 3).

### Species Composition Analysis

**FIGURE 4:**
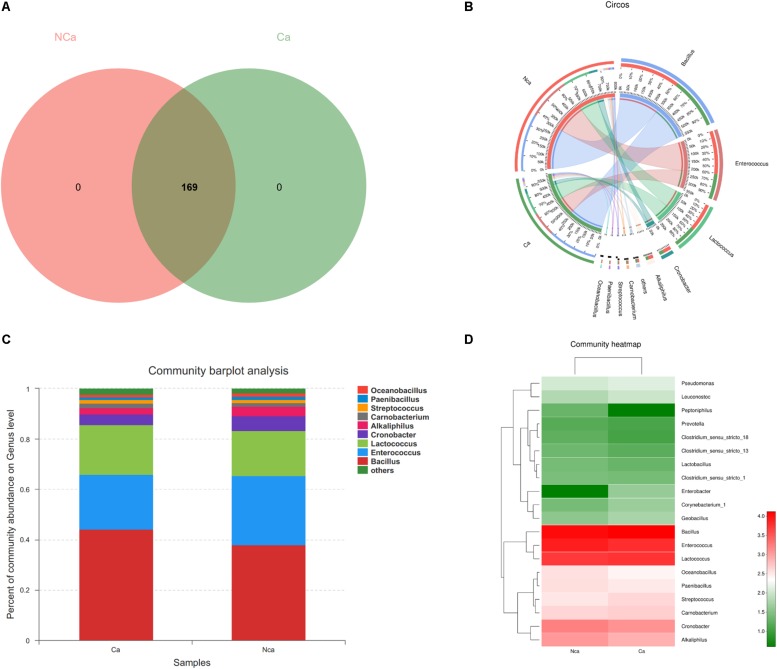
Species composition analysis. **(A)** The two groups did not show a specific composition. **(B)** The community pie chart shows the microbial composition of the two groups. **(C)** The community barplot for the two groups. **(D)** The heatmap demonstrates the compositions of the two groups.

The results show that there were no unique microbial species in the CA group or NCA group (Figure 4A). All samples consisted of *Oceanobacillus*, *Paenibacillus*, *Streptococcus*, *Carnobacterium*, *Alkaliphilus*, *Cronobacter*, *Lactococcus*, *Enterococcus*, *Bacillus*, and others (Figures 4B,C). However, there were some differences in species between the two groups (Figure 4D).

### Analysis of the Specific Microbial Species Differences

**FIGURE 5:**
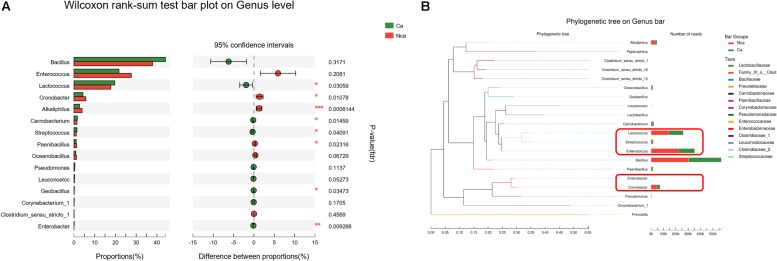
Analysis of the specific microbial species differences. **(A)** The differences in microbial species between the two groups were explored. **(B)** The evolution of the different microbial species in the two groups.

To further study the differences in microbial species between the two groups, we explored the differences in the specific microbial species between the two groups. The proportions of *Alkaliphilus*, *Enterobacter*, *Lactococcus*, *Cronobacter*, *Carnobacterium*, *Streptococcus*, *Paenibacillus*, and *Geobacillus* showed a significant difference between the CA group and the NCA group (*P* < 0.05, Figure 5A). In addition, some of the different microbial species in the two groups were evolutionarily similar, such as *Lactococcus*, *Streptococcus*, and *Enterococcus* (Figure 5B).

### Disease Status Discrimination With the Microbiome

**FIGURE 6:**
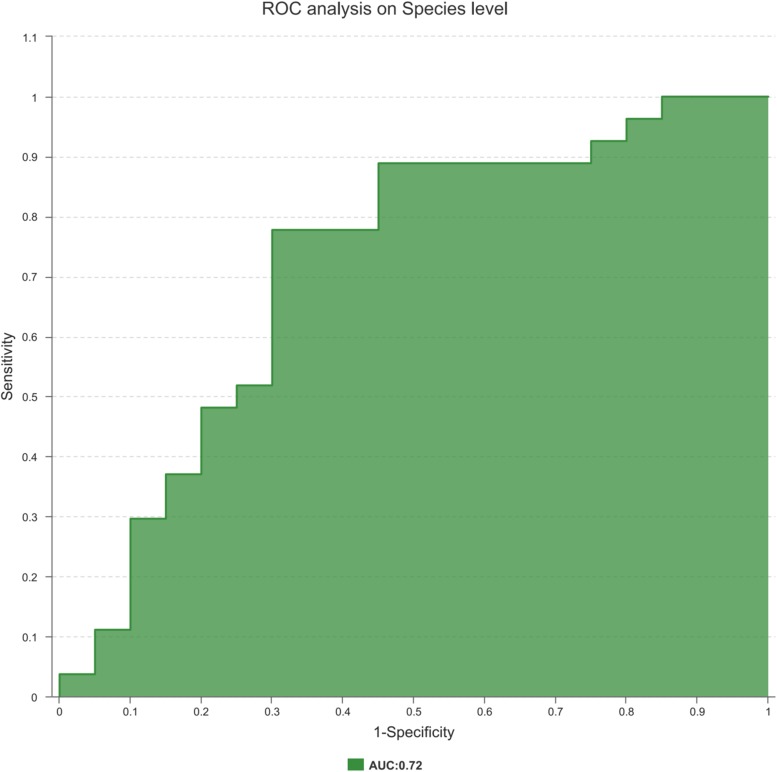
The ROC curve used to analyze the clinical accuracy of using differential bacteria obtained from the CA group and NCA group for the diagnosis of PCa.

Finally, to explore the potential ability of the prostate fluid microbiome to identify PCa status, we constructed a random forest model based on the prostate fluid microbiome signature composed of 8 associated genera. We constructed an ROC to analyse the clinical accuracy of using the gut microbiome for the diagnosis of PCa, and the AUC (area under the curve) was 0.72 (Figure 6), suggesting that it might play a role in the diagnosis of PCa.

## Discussion

PCa is one of the top malignancies in the male population worldwide. Although age, ethnicity, and family history are the risk factors for PCa, some other factors were recognized in the development and progression of PCa, such as infections of bacteria and virus, inflammation, and other environmental factors (Bosland, 1988; Maitland and Collins, 2008; Sutcliffe and Platz, 2008; Sfanos et al., 2013; Delcaru et al., 2017). All of above mentioned factors could influence the microbiological composition, the community of the fungi, viruses, bacteria and parasites living within the human body. These eventually impact human health and disease by interacting with each other and with the host (Holmes et al., 2011; Giorgetti et al., 2015; Pagliari et al., 2015). In recent years, the development of high throughput molecular-based methods for the identification of complex microbiomes has promoted the research in microbiome, which reveals the novel relationship between microbiological composition and human pathological conditions, such as colorectal cancer, PCa, diabetes and obesity (van Olden et al., 2015; Wang et al., 2015; Cavarretta et al., 2017; Komaroff, 2017; Liu et al., 2017).

In the present study, we revealed the microbiome of the prostatic fluid from PCa patients by using a massive 16S rRNA gene sequencing approach. In addition to confirming the existence of a prostate-specific microbiome, we also identified different microbial species between the CA group and the NCA group.

We collected prostatic fluid specimens from 32 patients with PCa and 27 non-PCa people, all participants with a high level of PSA. The rarefaction curves of all samples indicated that the depth of sequencing catered to the needs of 16S rRNA gene sequencing analysis. Then, we compared the microbial diversity of the CA and NCA groups by calculating the Shannon–Wiener index and Simpson diversity index. The results suggest that the NA group showed reduced microbial diversity when compared with the NCA group, demonstrating that microbial diversity may have a role in the progression of PCa. We further analyzed the specific microbial species in the two groups, but the results demonstrated that there were no idiosyncrasies in the microbial species in the CA group when compared with the NCA group. All samples consisted of *Oceanobacillus*, *Paenibacillus*, *Streptococcus*, *Carnobacterium*, *Alkaliphilus*, *Cronobacter*, *Lactococcus*, *Enterococcus*, *Bacillus*, and others. However, there were many differences of the abovementioned species between the two groups, such as *Alkaliphilus*, *Enterobacter*, *Lactococcus*, *Cronobacter*, *Carnobacterium*, *Streptococcus*, *Paenibacillus*, and *Geobacillus*. Nevertheless, some of the different microbial species in the two groups were evolutionarily similar, as shown in the phylogenetic tree.

In a recent prostate microbiome study, Riley et al. (1998) reported that 16S rDNA could be detected in prostate tissues with CPS at first. Ilaria Cavarretta et al. (2017) reported that the *Streptococcus* was almost exclusive presented in non-tumor regions, supporting the idea that the *Lactobacillales* might belong to a normal prostatic microbiome and might contribute to the balance of the host extracellular environment. In addition, in our study, the proportion of *Streptococcus* in the CA group was significantly higher than that in the NCA group. The difference might come from the sample type. The microbiome has been seen as a key factor in disease, controlling multiple pathways involved with metabolism (Shreiner et al., 2015). Some recent studies indicated that bile acids, lactate, fatty lipids and others could maintain endocrine functions to regulate tricarboxylic acid cycle, cholesterol, glucose and energy homeostasis via some bacteria (Swann et al., 2011; Serino et al., 2012; Hui et al., 2017). The host and its microbiota coproduce a great diversity of small molecules during metabolism, many of which might play critical roles in the occurrence and development of disease. In our study, some of the different microbial species in the two groups, such as *Lactococcus* spp. and *Streptococcus* spp., could change the environment through variety metabolites, which is thought to play a role in tumor growth.

This pilot study has several limitations. It is difficult to control for bacteria possibly introduced from the urinary tract, and the small sample number was a limitation of this study. A major innovation point of this analysis was that it provided the first detailed description of the microbiome environment in prostate fluid in CA and NCA patients.

## Conclusion

This 16S rRNA gene sequencing approach suggested novel findings of a large population of bacteria within the prostatic fluid, found differences in the microbial species in the prostatic fluid from non-PCa people and PCa patients, and demonstrated that bacteria may be beneficial in maintaining the stability of the microenvironment of the prostate, providing new ideas for the diagnosis of PCa with high PSA. Further studies might place more focus on clarifying the possible pathogenic role of bacteria and exploring the microbiome in other body fluids associated with PCa.

## Ethics Statement

Data collection followed the principles outlined in the Declaration of Helsinki; all participants had signed an informed consent document agreeing to deliver their own anonymous information for future studies. The study was approved by our local ethical committee (Renji/2013126).

## Author Contributions

XM and CC drafted the manuscript and designed the study. CC and LF carried out the DNA extraction and the sample collection. BD, XS, and SX collected the patients’ materials and clinical and patients’ information. XM and ML performed the sequencing. XM and WX performed the data interpretation, bioinformatical and statistical analyses, drafted the manuscript, and designed the study. All authors read and approved the final version of the manuscript.

## Conflict of Interest Statement

The authors declare that the research was conducted in the absence of any commercial or financial relationships that could be construed as a potential conflict of interest.

## Footnotes

1 http://drive5.com/uparse/

2 http://rdp.cme.msu.edu/
